# Evaluating Algorithmic Bias in 30-Day Hospital Readmission Models: Retrospective Analysis

**DOI:** 10.2196/47125

**Published:** 2024-04-18

**Authors:** H Echo Wang, Jonathan P Weiner, Suchi Saria, Hadi Kharrazi

**Affiliations:** 1 Bloomberg School of Public Health Johns Hopkins University Baltimore, MD United States; 2 Johns Hopkins Center for Population Health Information Technology Baltimore, MD United States; 3 Whiting School of Engineering Johns Hopkins University Baltimore, MD United States

**Keywords:** algorithmic bias, model bias, predictive models, model fairness, health disparity, hospital readmission, retrospective analysis

## Abstract

**Background:**

The adoption of predictive algorithms in health care comes with the potential for algorithmic bias, which could exacerbate existing disparities. Fairness metrics have been proposed to measure algorithmic bias, but their application to real-world tasks is limited.

**Objective:**

This study aims to evaluate the algorithmic bias associated with the application of common 30-day hospital readmission models and assess the usefulness and interpretability of selected fairness metrics.

**Methods:**

We used 10.6 million adult inpatient discharges from Maryland and Florida from 2016 to 2019 in this retrospective study. Models predicting 30-day hospital readmissions were evaluated: LACE Index, modified HOSPITAL score, and modified Centers for Medicare & Medicaid Services (CMS) readmission measure, which were applied *as-is* (using existing coefficients) and *retrained* (recalibrated with 50% of the data). Predictive performances and bias measures were evaluated for all, between Black and White populations, and between low- and other-income groups. Bias measures included the parity of false negative rate (FNR), false positive rate (FPR), 0-1 loss, and generalized entropy index. Racial bias represented by FNR and FPR differences was stratified to explore shifts in algorithmic bias in different populations.

**Results:**

The retrained CMS model demonstrated the best predictive performance (area under the curve: 0.74 in Maryland and 0.68-0.70 in Florida), and the modified HOSPITAL score demonstrated the best calibration (Brier score: 0.16-0.19 in Maryland and 0.19-0.21 in Florida). Calibration was better in White (compared to Black) populations and other-income (compared to low-income) groups, and the area under the curve was higher or similar in the Black (compared to White) populations. The retrained CMS and modified HOSPITAL score had the lowest racial and income bias in Maryland. In Florida, both of these models overall had the lowest income bias and the modified HOSPITAL score showed the lowest racial bias. In both states, the White and higher-income populations showed a higher FNR, while the Black and low-income populations resulted in a higher FPR and a higher 0-1 loss. When stratified by hospital and population composition, these models demonstrated heterogeneous algorithmic bias in different contexts and populations.

**Conclusions:**

Caution must be taken when interpreting fairness measures’ face value. A higher FNR or FPR could potentially reflect missed opportunities or wasted resources, but these measures could also reflect health care use patterns and gaps in care. Simply relying on the statistical notions of bias could obscure or underplay the causes of health disparity. The imperfect health data, analytic frameworks, and the underlying health systems must be carefully considered. Fairness measures can serve as a useful routine assessment to detect disparate model performances but are insufficient to inform mechanisms or policy changes. However, such an assessment is an important first step toward data-driven improvement to address existing health disparities.

## Introduction

### Background of Algorithmic Bias

Predictive algorithms and machine learning tools are increasingly integrated into clinical decision-making and population health management. However, with the increasing reliance on predictive algorithms comes a growing concern of exacerbating health disparities [1-3]. Evidence has shown that widely used algorithms that use past health care expenditures to predict high-risk patients have systematically underestimated the health care needs of Black patients [4]. In addition, studies have shown that predictive performances of models predicting intensive care unit mortality, 30-day psychiatric readmission, and asthma exacerbation were worse in populations with lower socioeconomic status [5,6].

With algorithmic bias as a potentially pervasive issue, a few checklists have been published to qualitatively identify and understand the potential biases derived from predictive models [7,8]. However, no agreed-upon quantitative method exists to routinely assess whether deployed models will lead to biased results and exacerbate health disparities faced by marginalized groups [2,9]. In this study, we define algorithmic bias as the differential results or performance of predictive models that may lead to differential allocation or outcomes between subgroups [10-12]. In addition, we define disparity as the difference in the quality of health care (the degree to which health services increase the likelihood of desired health outcomes) received by a marginalized population that is not due to access-related factors, clinical needs, preferences, and appropriateness of intervention [10,13]. Fairness metrics, which are a set of mathematical expressions that formalize certain equality between groups (eg, equal false negative rates [FNRs]), were proposed to measure and detect biases in machine learning models [12,14]. Although the machine learning community has shown that fairness metrics are a promising way to identify algorithmic bias, these metrics are criticized for being insufficient to reflect the heterogeneous and dynamic nature of health care [15,16]. Fairness metrics can also be misleading or conflicting due to their narrow focus on equal rates between groups [12,15]. Furthermore, these metrics could be interpreted without context-specific judgment or domain knowledge, thus failing to connect predictions to interventions and the downstream health care disparity [15,17]. Most importantly, these measures are often not fully tested in real-world predictive tasks and lack evidence on how well these measures’ interpretation could guide intervention planning.

### Background of Disparity in 30-Day Hospital Readmission

Predicting hospital readmissions is widely studied in health care management and delivery [18-21]. Hospital readmissions, especially unplanned or avoidable readmissions, are not only associated with a high risk of in-hospital mortality but also costly and burdensome to the health care system [19,22]. Since 2012, the Hospital Readmission Reduction Program by the Centers for Medicare & Medicaid Services (CMS) has imposed financial penalties for hospitals with excessive readmission rates [22]. CMS has consequently incentivized hospitals to segment patients by risk so that hospitals can target the delivery of these resource-intensive interventions to the patients at greatest risk, such as transitional care intervention and better discharge planning [19,23,24]. Many hospital readmission predictive models have been published, with >350 models predicting 30-day readmission identified in prior systematic reviews and our prior work [7,18,19,21,25]. The disparity in hospital readmission rates is well studied. For example, past studies have shown that Black patients have higher readmission rates after adjusting for demographic and clinical characteristics [26-29]. In addition to racial disparity, patients receiving care at racial and ethnic minority-serving hospitals [29,30] or living in disadvantaged neighborhoods have higher rates of readmission [31-33]. Research has also shown that disparity in health care use, including hospital readmission, is related to not only individuals’ racial and ethnic identity but also their communities [34]. Other research has also suggested that social environments, either the place of residence or the hospital where one receives care, may explain a meaningful portion of health disparity [35,36].

### Objectives

Despite model abundance and known disparity in hospital readmissions, research has been limited in evaluating how algorithmic bias or the disparate performances of these predictive models may impact patient outcomes and downstream health disparities once deployed. Lack of evidence is more prominent in how the model-guided intervention allocation may reduce or aggravate existing health disparities between different populations. To address this gap in evidence, in this study, we aimed to (1) implement a selection of fairness metrics to evaluate whether the application of common 30-day readmission predictive models may lead to bias between racial and income groups and (2) interpret the selected fairness metrics and assess their usefulness in the context of facilitating equitable allocation of interventions. In this paper, we represent the perspective of a health system or payer who uses an established, validated algorithm to identify patients at high risk of unplanned readmission so that targeted intervention can be planned for these patients. Thus, our main concern for algorithmic bias is the unequal allocation of intervention resources and the unequal health outcome as a result. Specifically, we are concerned about risk scores systematically underestimating or overestimating needs for a certain group, assuming the model we deploy is validated and has acceptable overall predictive performance.

## Methods

### Study Population and Data

This retrospective study included 1.9 million adult inpatient discharges in Maryland and 8.7 million inpatient discharges in Florida from 2016 to 2019. The State Inpatient Databases (SIDs) are maintained by the United States Agency for Healthcare Research and Quality, as part of the Healthcare Cost and Utilization Project (HCUP), were used for this analysis. The SIDs include longitudinal hospital care data in the United States, inclusive of all insurance payers (eg, Medicare, Medicaid, private insurance, and the uninsured) and all patient ages [37]. The SIDs capture >97% of all eligible hospital discharges in each state [38]. Maryland and Florida were selected due to their different population sizes, compositions (eg, racial and ethnic distribution and urban to rural ratio), and health care environment (Maryland’s all-payer model vs Medicaid expansion not adopted in Florida) [39,40]. In addition, Maryland and Florida are among a small subset of states in which the SIDs contain a “VisitLink” variable that tracks unique patients within the state and across years from 2016 to 2019, allowing for the longitudinal analysis of readmissions across hospitals and different calendar years [41]. The SIDs were also linked to the American Hospital Association’s Annual Survey Database to obtain hospital-level information. The study population excluded admissions where patients were aged <18 years, died in hospitals, were discharged against medical advice, or had insufficient information to calculate readmission (eg, missing the VisitLink variable or length of stay).

### Study Outcome

**Figure 1:**
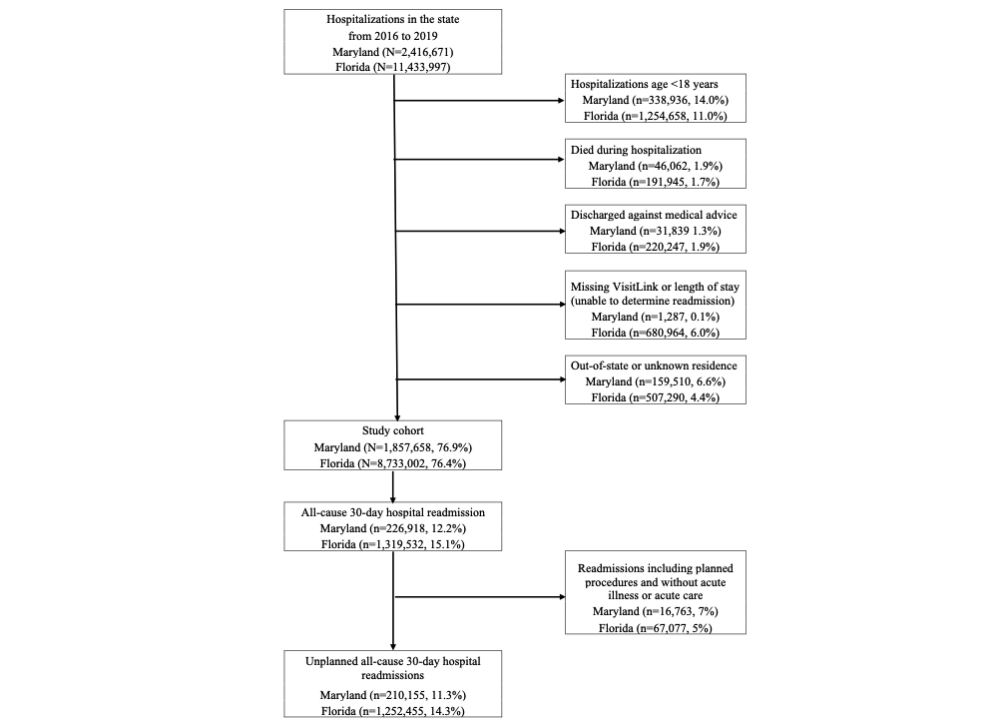
Determination of the study cohort and unplanned all-cause 30‐day readmission.

The calculation of 30-day readmission followed the definition used by the HCUP [42]. Any inpatient admission was counted as an index admission. The all-cause 30-day readmission rate was defined as the number of admissions with at least 1 subsequent hospital admission within 30 days, divided by the total number of admissions during the study period. Unplanned, all-cause 30-day hospital readmissions were identified using the methodology developed by CMS [43,44]. The study cohort selection process and determination of unplanned readmission are outlined in Figure 1.

### Predictive Models

The LACE index [45], the HOSPITAL score [46], and the CMS hospital-wide all-cause readmission measure [43] were included in the analysis as they were validated externally and commonly used in practice based on our prior review [7]. The LACE index and the HOSPITAL score were designed for hospital staff to identify patients at high risk of readmission for targeted intervention efforts and have been converted to a scoring system and extensively validated. Thus, the 2 models were applied to obtain the predicted risk scores without retraining, to mimic how the models were used in practice. In total, 2 of the HOSPITAL score predictors—low hemoglobin and low sodium levels at discharge—were not available in the SIDs, and thus were excluded. The total risk scores were adjusted as a result. Details of model variables and how the 2 models were implemented are reported in Multimedia Appendices 1 and 2. The CMS measure was evaluated using 2 approaches: applied *as-is* with existing coefficients and *retrained* to generate new coefficients using 50% of the sample. To ensure comparability between the CMS measure and other models, the predicted patient-level risk was used without the hospital-level effect from the original measure, and the CMS measure was limited to the “medicine cohort” [43]. On the basis of the CMS measure’s specification report, the patient population was divided into 5 mutually exclusive cohorts: surgery or gynecology, cardiorespiratory, cardiovascular, neurology, and medicine. The cohorts were determined using the Agency for Healthcare Research and Quality Clinical Classifications Software categories [43]. The medicine cohort was randomly split 50-50 into a retraining and testing data set. The CMS measure includes age and >100 variables, representing a wide range of condition categories. The measure was trained on the retraining data set with 5 cross-validations and then run on the testing data set using the new coefficients to obtain the performance and bias metrics for the CMS retrained model. Separately, the CMS measure with the published coefficients was run on the full medicine cohort data set to obtain performance and bias metrics for the CMS as-is model. The existing model thresholds were used to classify a positive, or high-risk, class: 10 points for LACE, and high-risk (5 in the adjusted scoring) for modified HOSPITAL. The optimal threshold identified using the Youden Index [47] on the receiver operating characteristic curve was used for the 2 CMS measures.

### Measures

We measured predictive performances and biases between Black and White subpopulations and between low-income and other-income subpopulations. Race is a normalized variable in the HCUP that indicates race and ethnicity. The low-income group was defined as the fourth quartile of the median state household income, whereas the remaining 3 quartiles were grouped as other income. The median state income quartiles were provided in HCUP SIDs and were calculated based on the median income of the patient’s zip code. Predictive performances of each model were derived for all population and each subpopulation using area under the curve (AUC), Brier statistic, and Hosmer-Lemeshow goodness of fit. Bias was represented by the group difference of the mathematical measures: false positive rate (FPR) difference (eg, FPR between Black and White patients), FNR difference, 0-1 loss difference, and generalized entropy index (GEI). FNR was calculated as the ratio between false negatives (those predicted as low risk while having an unplanned 30-day readmission) and the total number of positives. Similarly, the FPR was calculated as the ratio of false positives out of the total number of negative cases. Normalized total error rates is 0-1 loss, and it is calculated as the percentage of incorrect predictions. Bias measured by FPR, FNR, and 0-1 loss differences focus on unequal error rates. The GEI is a measure of income inequality and proposed to measure algorithm fairness between groups with a range between 0 and infinity, in which lower scores represent more equity [48].

### Ethical Considerations

This study was not human subjects research, as determined by the Johns Hopkins School of Public Health Institutional Review Board. No compensation was provided.

### Statistical Analysis

Primary analyses were conducted using R (version 4.0.2; R Foundation for Statistical Computing). The aggregate condition categories required to calculate unplanned readmission and CMS measures were calculated in SAS software (version 9.4; SAS Institute) using the programs provided by the agencies [49,50]. GEI measures were calculated using the AI Fairness 360 package published by IBM Corp [51]. The unit of analysis was admission. FNR and FPR results were first stratified by individual hospital and visualized in a scatter plot. The racial bias results were then stratified by hospital population composition (eg, percentage of Black patients), which was shown to associate with the overall outcome of a hospital [35]. Hospitals were binned by the percentage of Black patients served in a hospital (eg, >10% and >20%), and the racial bias measures with their 95% CIs were calculated for each bin. For FNR difference, FPR difference, and 0-1 loss difference, the distribution across 2 groups was calculated, and the significance of the measure difference was assessed using the Student *t* test (2-tailed) under the null hypothesis that the group difference was equal to 0. For all statistical tests, an α of .05 was used.

## Results

### Demographic and Clinical Characteristics

As presented in Table 1, among the 1,857,658 Maryland inpatient discharges from 2016 to 2019, a total of 55.41% (n=1,029,292) were White patients and 33.71% (n=626,280) were Black patients, whereas in Florida, 64.49% (5,632,318/8,733,002) of the inpatient discharges were White patients and 16.59% (1,448,620/8,733,002) were Black patients.

White patients in both states were older, more likely to be on private insurance, and less likely to reside in large metropolitan areas or be treated in major teaching or large hospitals in urban areas. Compared to White patients, Black patients in Maryland had a longer length of inpatient stay, more inpatient procedures, fewer inpatient diagnoses, higher inpatient charges, and more comorbidities and were more likely to be discharged to home or self-care. However, Black patients in Florida had fewer inpatient diagnoses, fewer procedures, and fewer total charges. These patients also had longer lengths of inpatient stays, more comorbidities, and were more likely to be discharged to home or self-care. In both Maryland and Florida, those in the lowest income quartile were younger, had a longer length of inpatient stay, had higher inpatient charges, had more comorbidities, and had fewer procedures than other-income groups. The low-income group was less likely to reside in metropolitan areas but was more likely to be treated in major teaching hospitals. Except for those noted in footnote c of Table 1, all characteristics showed statistically significant differences between racial and income groups (all *P* values <.001).

**Table 1:** Demographic characteristics by race and by income in Maryland (n=1,857,658) and Florida (n=8,733,002).

Characteristics and state			Race			Income	
			White	Black	Other	Low income	Other income
**Discharges, n (%)**							
	MD^a^		1,029,292 (55.41)	626,280 (33.71)	187,935 (10.12)	627,013 (33.75)	1,225,820 (65.99)
	FL^b^		5,632,318 (64.49)	1,448,620 (16.59)	1,598,392 (18.3)	2,600,326 (29.78)	6,028,720 (69.03)
**Age (y), mean (SD)**							
	MD		61.4 (19.9)	54.0 (19.4)	46.8 (20.7)	56.3 (19.8)	58.0 (20.8)
	FL		63.1 (19.4)	51.1 (19.8)	56.1 (21.6)	58.0 (20.4)	60.6 (20.5)
**Sex (female; yes), n (%)**							
	MD		586,641 (56.99)	377,063 (60.21)	128,138 (68.18)	364,718 (58.17)	733,135 (59.81)
	FL		3,050,611 (54.16)	856,343 (59.11)	942,996 (59)	1,454,239 (55.93)	3,372,155 (55.93)^c^
**Payer, n (%)**							
	**Medicare**						
		MD	550,364 (53.47)	262,512 (41.92)	43,461 (23.13)	293,009 (46.73)	566,503 (46.21)
		FL	3,386,956 (60.13)	589,574 (40.7)	715,483 (44.76)	1,358,285 (52.24)	3,301,163 (54.76)
	**Medicaid**						
		MD	142,138 (13.81)	192,443 (30.73)	71,450 (38.02)	192,624 (30.72)	215,530 (17.58)
		FL	504,531 (8.96)	373,896 (25.81)	315,306 (19.73)	505,805 (19.45)	682,879 (11.33)
	**Private**						
		MD	306,929 (29.82)	148,781 (23.76)	59,381 (31.6)	120,346 (19.19)	398,459 (32.51)
		FL	1,183,304 (21.01)	287,528 (19.85)	398,712 (24.94)	422,946 (16.27)	1,439,482 (23.88)
**Residence (large metropolitan), n (%)**							
	MD		839,688 (81.58)	586,868 (93.72)	178,015 (94.71)	460,587 (73.46)	1,154,341 (94.17)
	FL		2,939,039 (52.18)	1,027,033 (70.9)	1,319,976 (82.58)	1,530,136 (58.84)	3,738,731 (62.02)
**Length of stay, mean (SD)**							
	MD		4.73 (6.10)	5.22 (7.20)	4.19 (6.22)	5.17 (6.89)	4.68 (6.32)
	FL		4.96 (6.37)	5.24 (7.98)	4.76 (6.76)	5.16 (7.26)	4.89 (6.51)
**Total charges, mean (SD)**							
	MD		17,000 (22,700)	17,800 (25,800)	14,600 (23,000)	18,000 (25,100)	16,500 (23,200)
	FL		68,500 (88,800)	62,800 (95,900)	68,900 (100,000)	67,300 (93,600)	67,800 (92,000)
**Discharge type (home or self-care), n (%)**							
	MD		780,238 (75.8)	490,051 (78.25)	165,538 (88.08)	486,882 (77.65)	956,510 (78.03)
	FL		4,408,065 (78.26)	1,240,314 (85.62)	1,367,677 (85.57)	2,102,035 (80.84)	4,873,457 (80.84)^c^
**CCI^d^ score, mean (SD)**							
	MD		0.498 (1.04)	0.594 (1.18)	0.359 (0.962)	0.573 (1.13)	0.486 (1.06)
	FL		0.516 (1.04)	0.616 (1.23)	0.529 (1.13)	0.575 (1.13)	0.516 (1.07)
**Number of procedures, mean (SD)**							
	MD		1.68 (2.44)	1.71 (2.60)	2.00 (2.38)	1.69 (2.59)	1.74 (2.44)
	FL		1.57 (2.34)	1.50 (2.34)	1.57 (2.31)	1.49 (2.33)	1.59 (2.33)
**Number of diagnoses, mean (SD)**							
	MD		15.5 (8.20)	14.6 (7.84)	11.1 (7.07)	15.3 (8.04)	14.4 (8.08)
	FL		13.3 (7.33)	11.7 (7.19)	10.8 (6.91)	12.6 (7.33)	12.5 (7.29)
**Hospital type (major teaching; yes), n (%)**							
	MD		181,493 (17.63)	120,649 (19.26)	27,808 (14.80)	172,092 (27.45)	158,693 (12.95)
	FL		576,819 (10.24)	231,379 (15.97)	242,754 (15.19)	354,901 (13.65)	691,635 (11.47)
**Hospital beds (≥200 beds), n (%)**							
	MD		741,956 (72.08)	460,667 (73.56)	150,745 (80.21)	482,604 (76.97)	878,817 (71.69)
	FL		3,546,967 (62.98)	981,735 (67.77)	967,036 (60.5)	1,735,704 (66.75)	3,730,066 (61.87)
**Urban hospital (yes), n (%)**							
	MD		943,928 (91.71)	602,779 (96.25)	179,091 (95.29)	548,230 (87.44)	1,187,298 (96.86)
	FL		2,859,038 (50.76)	858,993 (59.3)	903,705 (56.54)	1,340,735 (51.56)	3,256,784 (54.02)

^a^MD: Maryland.

^b^FL: Florida.

^c^*P* values were computed between racial groups and between income groups, respectively. All *P* values are <.001 except for the ones in this footnote: *P* value for female between income groups=.80 and for discharge type between income groups=.99.

^d^CCI: Charlson Comorbidity Index.

### Predictive Performance

The observed 30-day unplanned readmission rates in Maryland were higher in the Black and low-income patient groups (ie, 11.13% for White patients, 12.77% for Black patients, 10.59% for other-income patients, and 12.73% for low-income patients; Table 2).

**Table 2:** Observed and predicted 30-day unplanned readmission rates by model and state.

			Observed (%)					Predicted^a^ (%)																		
			MD (n=1,857,658)		FL (n=8,733,002)		LACE^b^						HOSPITAL^c^					CMS^d^ as-is					CMS retrained			
							MD^e^ (n=1,857,658)			FL^f^ (n=8,733,002)		MD (n=1,857,658)			FL (n=8,733,002)		MD (n=714,917)			FL (n=2,636,671)		MD (n=357,458)				FL (n=1,318,335)
Total			11.31		14.34		14.48			15.88		12			14.61		10.48			10.26		14.71				16.5
**Race**																										
	White	11.13		13.94		12.92			14.81		11.62			14.07		10.27			10.11		14.14			15.97		
	Black	12.77		17.14		18.62			21.33		14.12			18.26		10.82			10.85		15.84			18.93		
**Income**																										
	Other	10.59		13.6		12.88			14.62		10.84			13.66		10.37			10.19		14.3				16.17	
	Low	12.73		16.03		17.6			18.72		14.29			16.77		10.67			10.43		15.46				17.25	

^a^Predicted: the predicted readmission rates for LACE and HOSPITAL were calculated as the percentage of patients at high risk of unplanned readmission based on the model output for the group; and the predicted readmission rates for the two CMS models were the predicted probability of being at high risk of unplanned readmission for the group.

^b^LACE: The LACE Index for readmission risk.

^c^HOSPITAL: The modified HOSPITAL score for readmission risk.

^d^CMS: Centers for Medicare & Medicaid Services (readmission measure).

^e^MD: Maryland.

^f^FL: Florida.

A fair and well-calibrated predictive model would be assumed to overpredict or underpredict readmission rates to a similar degree across racial or income groups. Compared to the observed readmission rates, the LACE index overestimated readmission rates in all subpopulations and was more pronounced in Black and low-income populations. The readmission rates estimated by the modified HOSPITAL score were closest to the observed rates. The CMS as-is model underestimated across subpopulations, and the estimated rates of readmission were similar between subpopulations, while the retrained CMS model overestimated in all subpopulations to a similar degree. In Florida, the observed 30-day unplanned readmission rates were higher than those in Maryland in all populations. Similar to Maryland, Florida’s observed readmission rates were also higher in the Black and low-income groups (ie, 13.94% for White populations, 17.14% for Black populations, 13.6% for other-income populations, and 16.03% for low-income populations) and had similar overestimation and underestimation patterns (Table 2).

As presented in Table 3, in Maryland, the retrained CMS model had better predictive performance (AUC 0.74 in all subpopulations) than the other 3 models, which only achieved moderate predictive performance (AUC between 0.65 and 0.68). The modified HOSPITAL score had the best calibration (Brier score=0.16−0.19 in all subpopulations), whereas the CMS as-is model performed poorly on the Brier score. Calibration was better in the White (compared to the Black) population and other-income (compared to low-income) populations in both states, and the AUC was higher or similar in the Black (compared to the White) population. In Florida, the CMS retrained model also performed better than the other models in all subpopulations (AUC 0.68-0.72), and the modified HOSPITAL score had the best calibration (Brier score 0.19-0.21). All models demonstrated excellent goodness of fit across subpopulations (Table 3).

**Table 3:** Predictive performances of each 30-day readmission model in Maryland and Florida.

All vs group and performance measure					LACE^a^					HOSPITAL^b^					CMS^c^ as-is					CMS retrained		
					MD^d^		FL^e^		MD			FL		MD			FL		MD			FL
**All**																						
	AUC^f^			0.68		0.68		0.65			0.66		0.66			0.65		0.74			0.69	
	Brier statistic			0.19		0.21		0.17			0.20		0.44			0.37		0.32			0.32	
	Hosmer-Lemeshow, *P* value			<.001		<.001		<.001			<.001		<.001			<.001		<.001			<.001	
**Group**																						
	**Race**																					
		**White**																				
			AUC	0.68		0.67		0.64			0.65		0.65			0.63		0.74			0.68	
			Brier statistic	0.18		0.21		0.17			0.20		0.41			0.36		0.31			0.32	
			Hosmer-Lemeshow, *P* value	<.001		<.001		<.001			<.001		<.001			<.001		<.001			<.001	
		**Black**																				
			AUC	0.68		0.68		0.67			0.69		0.67			0.68		0.74			0.72	
			Brier statistic	0.22		0.25		0.19			0.21		0.49			0.43		0.33			0.36	
			Hosmer-Lemeshow, *P* value	<.001		<.001		<.001			<.001		<.001			<.001		<.001			<.001	
	**Income**																					
		**Other**																				
			AUC	0.69		0.68		0.65			0.66		0.65			0.64		0.74			0.69	
			Brier statistic	0.17		0.20		0.16			0.19		0.43			0.36		0.31			0.32	
			Hosmer-Lemeshow, *P* value	<.001		<.001		<.001			<.001		<.001			<.001		<.001			<.001	
		**Low**																				
			AUC	0.68		0.67		0.66			0.67		0.67			0.66		0.74			0.70	
			Brier statistic	0.21		0.23		0.19			0.21		0.47			0.39		0.33			0.34	
			Hosmer-Lemeshow, *P* value	<.001		<.001		<.001			<.001		<.001			<.001		<.001			<.001	

^a^LACE: The LACE Index for readmission risk.

^b^HOSPITAL: The modified HOSPITAL score for readmission risk.

^c^CMS: Centers for Medicare & Medicaid Services (readmission measure).

^d^MD: Maryland.

^e^FL: Florida.

^f^AUC: area under the curve.

### Bias Measures

Misclassification rates (ie, FPR difference and FNR difference) indicate relative between-group bias, whereas 0-1 loss differences indicate the overall error rates between groups. The between-group GEI indicates how unequally an outcome is distributed between groups [48]. In Maryland, the retrained CMS model and the modified HOSPITAL score had the lowest racial and income bias (Table 4).

Specifically, the modified HOSPITAL score demonstrated the lowest racial bias based on 0-1 loss, FPR difference, and GEI, and the lowest income bias based on FPR and GEI. Retrained CMS demonstrated the lowest racial bias based on 0-1 loss and FNR difference, and the lowest income bias on all 4 measures. In Florida, racial biases based on FPR and FNR differences was generally greater than that in Maryland, especially for FNR differences. In Florida, the modified HOSPITAL score showed the lowest racial bias based on 0-1 loss, FPR difference, and GEI; the LACE index showed the lowest racial bias in FNR difference. Each model scored the best in at least one measure of income bias, but the overall HOSPITAL score and retrained CMS showed the lowest income bias in Florida. In both states, the White and other-income patient groups had a higher FNR, indicating that they were more likely to be predicted as low risk while having a 30-day unplanned readmission. The Black and low-income patient groups had a higher FPR, indicating that they were more likely to be predicted to be high-risk and not have a 30-day unplanned readmission. The overall error rates were higher in the Black and low-income patient groups compared to the White and other-income patient groups, respectively. Except for GEI and the values noted with a footnote in Table 4, all other measures showed statistically significant differences (all *P* values <.001) between racial and income groups, respectively.

**Table 4:** Bias measures of evaluated 30-day readmission models in Maryland and Florida.

Measures and state				All		White		Black		Difference (B-W)^a^		Other income		Low income		Difference (L-O)^a^
**LACE^b^**																
	**Maryland**															
		0-1 loss	0.19		0.18		0.22		0.04		0.17		0.21		0.04	
		FNR^c^	0.69		0.72		0.63		−0.10^d^		0.71		0.65		−0.06	
		FPR^e^	0.12		0.11		0.16		0.05		0.11		0.15		0.04	
		GEI^f^ (between-group)	N/A^g^		N/A		N/A		0.03		N/A		N/A		0.02	
	**Florida**															
		0-1 loss	0.21		0.21		0.25		0.04		0.20		0.23		0.03	
		FNR	0.68		0.71		0.60		−0.11		0.70		0.64		−0.06	
		FPR	0.13		0.12		0.18		0.05		0.12		0.16		0.04	
		GEI (between-group)	N/A		N/A		N/A		0.02		N/A		N/A		0.01	
**HOSPITAL^h^**																
	**Maryland**															
		0-1 loss	0.17		0.17		0.19		0.02		0.16		0.19		0.03	
		FNR	0.73		0.75		0.69		−0.07		0.75		0.69		−0.06	
		FPR	0.10		0.10		0.12		0.02		0.10		0.12		0.03	
		GEI (between-group)	N/A		N/A		N/A		0.01		N/A		N/A		0.01	
	**Florida**															
		0-1 loss	0.20		0.20		0.21		0.01		0.19		0.21		0.02	
		FNR	0.68		0.71		0.59		−0.12		0.71		0.64		−0.07	
		FPR	0.12		0.12		0.14		0.02		0.11		0.14		0.02	
		GEI (between-group)	N/A		N/A		N/A		0.01		N/A		N/A		0.01	
**CMS^i^ (as-is)**																
	**Maryland**															
		0-1 loss	0.44		0.41		0.49		0.07		0.43		0.47		0.04	
		FNR	0.30		0.34		0.24		−0.10		0.32		0.26		−0.06	
		FPR	0.47		0.43		0.54		0.11		0.45		0.51		0.06	
		GEI (between-group)	N/A		N/A		N/A		0.05		N/A		N/A		0.02	
	**Florida**															
		0-1 loss	0.37		0.36		0.43		0.08		0.36		0.39		0.02	
		FNR	0.42		0.47		0.28		−0.18		0.44		0.38		−0.06	
		FPR	0.36		0.34		0.47		0.13		0.35		0.40		0.04	
		GEI (between-group)	N/A		N/A		N/A		0.05		N/A		N/A		0.02	
**CMS (retrained)**																
	**Maryland**															
		0-1 loss	0.32		0.31		0.33		0.02		0.31		0.33		0.02	
		FNR	0.29		0.31		0.26		−0.05		0.30		0.27		−0.03^d^	
		FPR	0.32		0.31		0.35		0.04		0.31		0.35		0.03	
		GEI (between-group)	N/A		N/A		N/A		0.02		N/A		N/A		0.01	
	**Florida**															
		0-1 loss	0.32		0.32		0.36		0.04		0.32		0.34		0.02	
		FNR	0.38		0.41		0.28		−0.13		0.40		0.35		−0.05^d^	
		FPR	0.31		0.30		0.38		0.08		0.31		0.34		0.04	
		GEI (between-group)	N/A		N/A		N/A		0.03		N/A		N/A		0.01	

^a^The columns Difference (B-W) and Difference (L-O) indicate algorithmic bias measured as the difference in the bias measure (eg, FNR and FPR) between Black and White patients and between low-income and other-income groups.

^b^LACE: The LACE Index for readmission risk.

^c^FNR: false negative rate.

^d^All *P* values of the bias measures are <.001 except for the ones in this footnote: the *P* value for FNR difference of LACE in MD is .41, and the FNR difference of CMS retrained in MD is .45, and FNR difference of CMS retrained in FL is .005. Statistical tests were not conducted for the GEI as this measure produces one value for the population.

^e^FPR: false positive rate.

^f^GEI: generalized entropy index.

^g^N/A: not applicable.

^h^HOSPITAL: The modified HOSPITAL score for readmission risk.

^i^CMS: Centers for Medicare & Medicaid Services (readmission measure).

### Stratification Analyses

**Figure 2:**
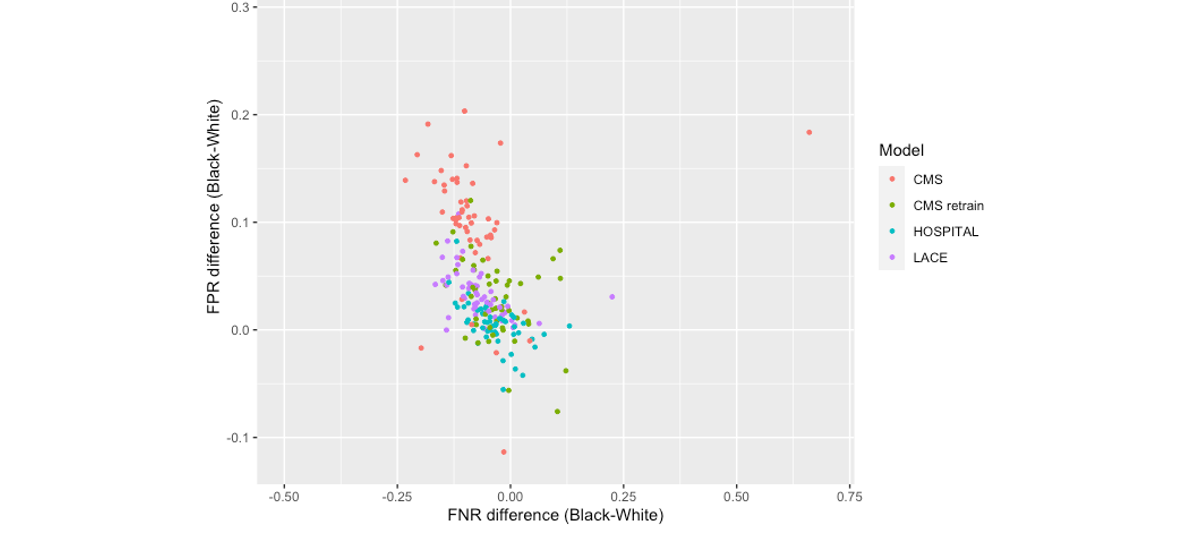
Measures of racial bias in predicting readmission across individual hospitals in Maryland. CMS: Centers for Medicare & Medicaid Services; FNR: false negative rate; FPR: false positive rate.

**Figure 3:**
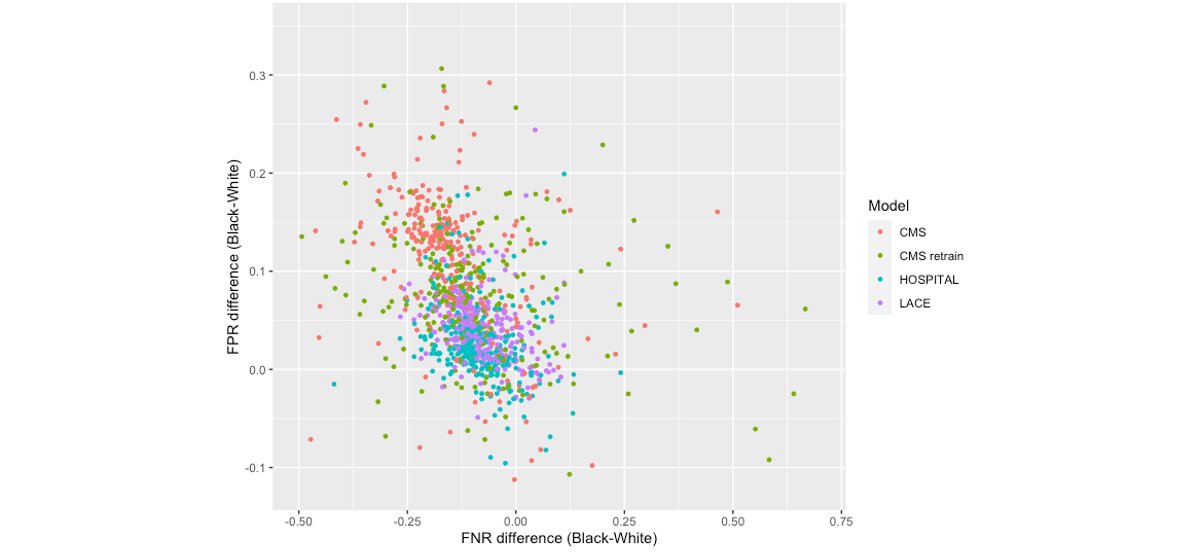
Measures of racial bias in predicting readmission across individual hospitals in Florida. CMS: Centers for Medicare & Medicaid Services; FNR: false negative rate; FPR: false positive rate.

The results were first stratified by hospital and then by patient population composition (percentage of Black patients). As shown in Figure 2, the models’ FNR differences and FPR differences between the Black and White patients varied by hospital within the state, indicating hospital shifts when applying the same model. The modified HOSPITAL score was more likely to cluster near the “equality lines” (ie, when the FNR or FPR difference is 0) than other models in both states. Colors representing LACE and CMS as-is were mostly distributed in the first quadrant in Maryland, indicating that the majority of hospitals had a positive FPR difference (ie, Black patients with higher FPR) and a negative FNR difference (ie, White patients with higher FNR) when applying these 2 models (Figure 2). Despite most hospitals falling in the first quadrant, the variance between hospitals appeared to be greater in Florida (Figure 3). In addition, more hospitals in Florida fell in the far corners of the first and fourth quadrants than those in Maryland, indicating more hospitals with severe bias (eg, large racial differences in FPR or FNR). Refer to Multimedia Appendix 3 for the measures of income bias and hospital distribution for Maryland and Florida.

**Figure 4:**
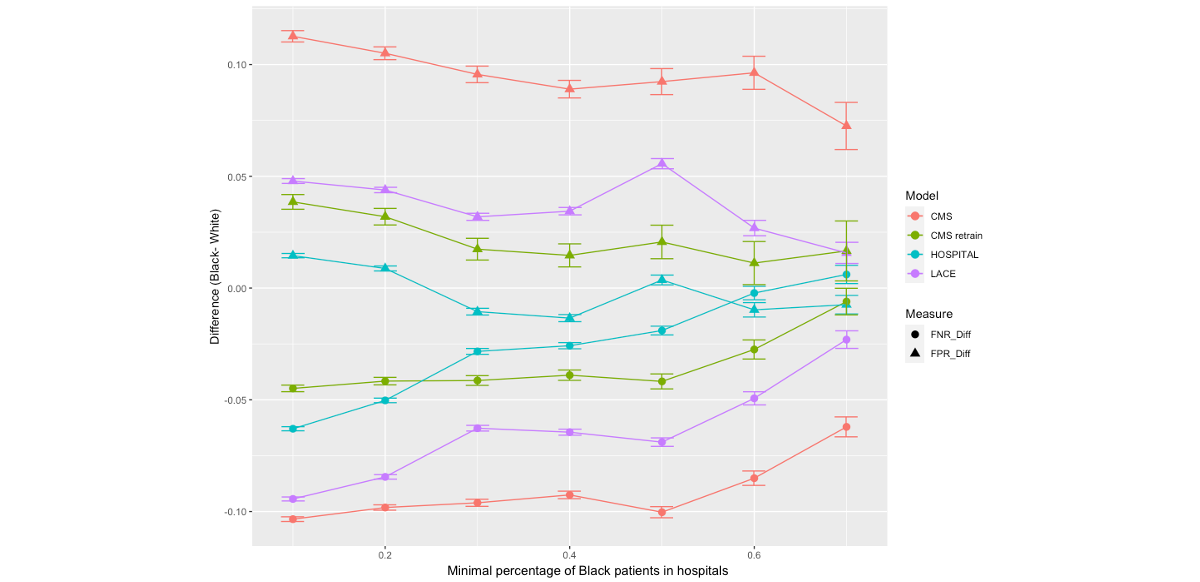
Bias measures by hospital patient population composition (percentage of Black patients) in Maryland. CMS: Centers for Medicare & Medicaid Services; FNR: false negative rate; FPR: false positive rate.

**Figure 5:**
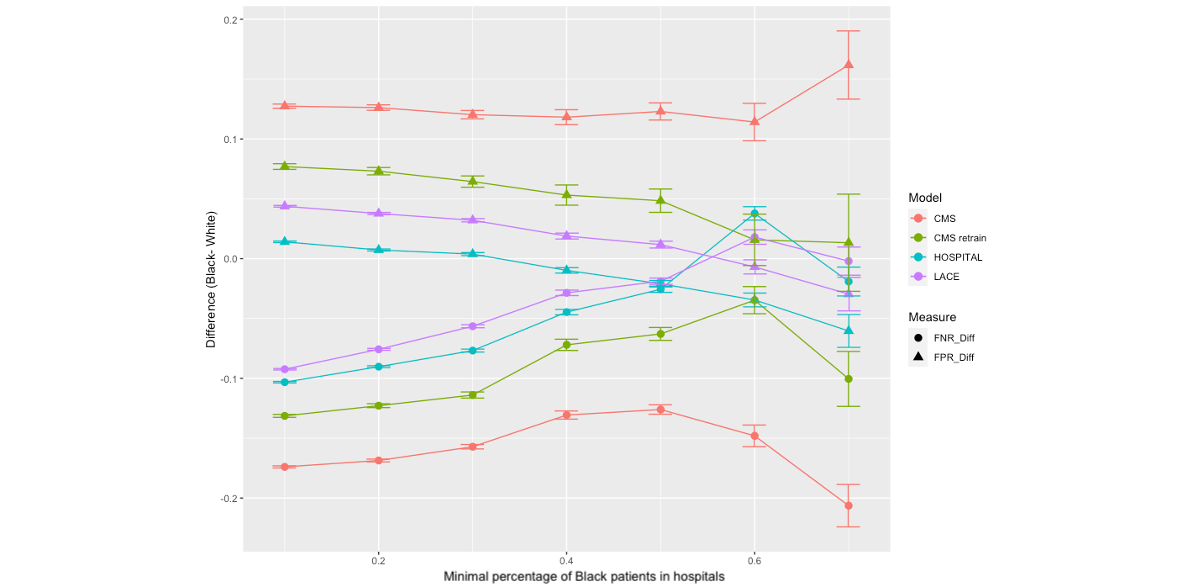
Bias measures by hospital patient population composition (a percentage of Black patients) in Florida. CMS: Centers for Medicare & Medicaid Services; FNR: false negative rate; FPR: false positive rate.

Hospitals with a higher percentage of Black patients have been shown to be associated with low resources and poorer outcomes for their patients [35]; thus, the results were stratified by the proportion of Black patients served in a hospital. In Figures 4 and 5, each data point represents the racial bias (FNR difference or FPR difference) in a stratum of hospitals with a certain percentage of Black patients (eg, hospitals with at least 20% of Black patients). The error bars show the 95% CI of the bias measure in the strata. In both figures, the racial biases of all models, represented as FNR and FPR differences, decreased and approached zero as the hospital population became more diverse. In Maryland, the diminishing racial bias was particularly notable in hospitals where >50% of patients were Black (Figure 4). The diminishing racial bias was also observed in Florida’s hospitals (Figure 5). The direction of bias flipped for the LACE index and the modified HOSPITAL score in Florida hospitals with >50% of Black patients. In hospitals with a lower percentage of Black patients, Black patients had a lower FNR compared to White patients, while in hospitals with a higher percentage of Black patients, White patients had a higher FNR (Figure 4). In Florida, the widening gap shown in the 2 CMS models for hospitals serving >60% of Black patients was likely attributed to the small number of hospitals and small sample size in the strata (Figure 5). Refer to Multimedia Appendix 4 for the details on the bias measures stratified by payers for both Maryland and Florida.

## Discussion

### Overall Findings

The abundance of research on fairness and bias has provided potential means to quantify bias, but there has been a gap to operationalize these metrics, interpret them in specific contexts, and understand their impact on downstream health disparity [7]. Our analysis demonstrated a practical use case for measuring algorithmic bias when applying or deploying previously validated 30-day hospital readmission predictive models in a new setting. Our approach to testing the fairness measures could serve as a framework for routine assessment of algorithmic bias for health care predictive models, and our results also revealed the complexity and limitations of using mathematical bias measures. According to these bias measures, the retrained CMS model and the modified HOSPITAL score showed the best predictive performance and the lowest bias in Maryland and Florida. However, the CMS as-is model showed subpar performance in both states, indicating that retraining on the local data not only improved predictive performance but also reduced group bias. In addition, large variations were detected between hospitals, and system- or hospital-level factors needed to be considered when interpreting algorithmic bias.

### Measure Interpretation

Caution must be taken when using algorithmic bias to guide equitable intervention allocation, as the bias measures may not include key context. When designing a risk-based intervention based on model output, we would be naturally more concerned about FNR, as a higher FNR means a groups that is more likely to be predicted low in risk of readmission will indeed be readmitted, indicating missed opportunities for intervention [52]. Looking at bias measures alone, our results suggest that the risk to White and higher-income patients has a systematically higher proportion of false negatives estimated by common readmission models, suggesting more missed opportunities to intervene and prevent unplanned readmissions. This observation is contrary to our assumption, and other parts of the results show that White and higher-income patients were less sick with lower readmission rates. An explanation would suggest that the higher FNR observed in the White and higher-income patient groups might be attributed to health care use patterns. For example, research has shown that White individuals and higher socioeconomic patient groups were more likely to overuse health care resources, while Black patients and disadvantaged groups tended to underuse them [53-55]. The overutilizers could have more unplanned visits to the hospital when the risk was not high, while the underusing group may be more likely to defer or skip care and only use costly hospital resources when they must. Similarly, a higher FPR in Black and low-income patient groups would indicate more wasted resources on “false positives.” However, such a conclusion did not align with the rest of the study findings. These subpopulations, on average, had more chronic comorbidities and longer inpatient stays, indicating that Black and low-income patient groups were more likely to have conditions that warrant an unplanned readmission but did not show up in the observed data, potentially alluding to a health care access gap in these groups. In this case, drawing a conclusion simply based on the face value of higher FPR would lead to a reduction in the resources allocated to the sicker, more vulnerable populations. It is also important to note that, despite the racial difference in health behaviors and outcomes, race merely represents a social classification rather than the driver of the observed differences [56]. Although the performance of the evaluated readmission models differed by race, we do not recommend including race as a variable in a predictive model unless race is a biological or clinical risk factor for the predictive outcome.

The interpretation of measurable bias requires considering models’ predictive performance, the nature of health data, analytic frameworks, and the underlying health care delivery system. In our analysis, all models had modest performance, and the high FNRs may deter their application in a real setting, especially in the score-based models of LACE and HOSPITAL (ie, FNR ranges from 0.63 to 0.75). When calculating these measures, we assumed the observed outcome (ie, 30-day unplanned readmission) as the ground truth; however, it was important to recognize the key limitations of this truth and the measured bias. First, despite the HCUP state inpatient data being one of the most comprehensive and high-quality data for studying readmission, no guarantee existed that all readmissions and their causes were captured. It is possible that a patient had conditions that warranted an unplanned revisit to the hospital but either did not occur due to the patient’s unwillingness to seek treatment in time [57,58] or did not get documented (eg, out-of-state admissions were not captured in HCUP’s state-wide inpatient data by design). Such underdocumentation was more likely to impact disadvantaged populations and those with fragmented care, thus introducing embedded bias into the underlying data. Second, a higher percentage of Black patients sought care in academic teaching institutions (eg, 120,649/626,280, 19.26% of Black patients in Maryland and 231,379/1,448,620, 15.97% of Black patients in Florida, compared to 181,493/1,029,292, 17.63% of White patients in Maryland and 576,819/5,632,318, 10.24% of White patients in Florida), which were generally considered to deliver high-quality care [35,59,60]. These hospitals may have a more effective readmission prevention program while serving sicker patients, contributing to a higher FPR among Black and low-income patients. Third, as shown in Figure 2, we observed that hospitals that served a high proportion of Black patients had a lower algorithmic bias. For example, in Maryland, the majority Black hospitals (>70% of patients served are Black) were in resource-poor neighborhoods, and both White and Black patients had similar higher-than-average readmission rates in these hospitals (data not shown). The fairer model performance in these hospitals was not necessarily a reflection of a higher quality of care, as all patients served in those hospitals had higher unplanned readmission rates. Finally, whether a readmission was unplanned or planned was determined using a well-established algorithm developed by CMS [43,44], which categorized readmissions based on the nature of the diagnoses and procedures (eg, acute vs routine). Research demonstrated that different diagnosis intensities existed between regions and hospitals, and a higher intensity of services was associated with a higher prevalence of common chronic diseases [61]. If diagnosis was not just a patient attribute but indeed reflected the systematic characteristics of the health care environment [62], the quality of unplanned readmission classification and other predictors in our models would be subject to encoded bias in the health care system. In fact, in our population, the average number of diagnoses was higher in White patients than in Black patients and higher in Maryland than in Florida, indicating the presence of such systematic variation (Table 1). Of course, this is not a unique issue with our data set; electronic health records and other health data sets also reflect histories of unequal access to health care and carry racial, ethnic, socioeconomic, and other societal biases due to how the data are collected [2,3,63].

### Utility of Bias Measures

Once the limitations of real-world health data are acknowledged, the expectation of equity and interpretation of the measurable bias should adjust accordingly. First, it will be too restrictive to expect mathematical equality for measurable bias; rather, it is best viewed as a relative value to aid in the selection of a less biased model. Most real-world problems are based on imperfect data, and pushing the model to perform equally on these measures will inevitably create unintended results (eg, sacrificing accuracy and potentially increasing bias for other subpopulations) [15]. Second, a validated and accurate model may reveal the gap between the “supposed-to-be” state and the reality in the underlying data, showing areas of unmet needs [16,64], as we observed in our Black and low-income populations. Finally, the bias measures alone provide limited evidence about which group is being biased against and in which way. A conclusion based solely on the face value of a few bias measures can be misleading and may exacerbate the disparity already faced by marginalized groups. These quantitative bias measures are useful to evaluate a model’s disparate group performance on a given data set, but they are insufficient to inform the intervention allocation or mechanisms of potential bias, which are key to the mitigation strategies [15]. In addition, our study did not evaluate other definitions of bias, such as calibration or predictive parity, which do not focus on error rates and may require unique interpretation considerations.

This analysis addressed a fundamental gap in operationalizing fairness techniques. The selection of a bias definition and appropriate bias measures is as important as detecting bias itself, yet it has remained a blind spot in practice [2]. In addition to the fact that these mathematical notions cannot be satisfied simultaneously, using the appropriate measures is also highly contextual and data dependent [65,66]. For example, having a model with equal positive predictions across groups (known as demographic or statistical parity) would not be a meaningful measure for inherently unbalanced outcomes such as 30-day readmissions; however, based on the fairness concept, satisfying any of the bias measures would mean a fair model. In this study, the 4 evaluated bias measures showed consistent results, despite each measuring a different definition of bias. All selected measures were able to demonstrate the magnitude of bias, but FNR and FPR differences were the most informative, as they indicated the direction of bias and were more interpretable in the context of mitigation actions. In our attempt to translate the algorithmic bias findings to intervention planning, we found that the bias measures could serve as a quick and routine assessment to compare algorithms, subpopulations, or localities (eg, hospitals) to help target further investigation of drivers of potential disparity. However, simply relying on these statistical notions to make decisions could obscure or underplay the causes of health care disparities, and a more comprehensive approach is necessary. In real-world applications, the practical goal of predictive modeling must incorporate predictive accuracy and algorithmic bias, among other operational considerations. As there is usually a trade-off between these 2 model performance goals, the best model is likely the one that balances the 2 goals rather than the one achieving the highest possible accuracy or fairness alone.

### Limitations

Our analysis has several limitations and caveats. First, none of the models evaluated in this analysis had high accuracy, which may affect the measurement of misclassifications. For simplicity and the focus on interpreting the bias measures, we did not evaluate machine learning models that usually improve local accuracy [20]. While the LACE index and the HOSPITAL score were used by hospitals to manage readmissions, the CMS measure was mostly used in payer operations or population health management in addition to CMS purposes (eg, budget allocation and hospital penalties); thus, it was not used as a typical predictive model. Although we believe the models evaluated in this study represented practical scenarios, we were unable to assess if a particular type of models, variables, weights, or modeling structures were more likely to be algorithmically biased. Second, we did not evaluate the scenario in which models can be optimized to minimize and constraint bias during training or retraining. Model optimization has been a popular approach to developing fair models but, it was considered out of scope as this analysis focused on model application and bias identification. Third, we only included bias measures that are algorithm-agnostic and can be routinely calculated; thus, they were not comprehensive or exclusive. Fourth, the conclusion was based on Maryland and Florida data, which would not represent all states nor the national average. For example, Maryland is a small state with an all-payer model payment system [39] and a high percentage of patients seeking care in neighboring states, whereas Florida is a large state with a large Hispanic population and has not adopted Medicaid expansion [40]. In addition, the data set we used was administrative in nature and did not have the detailed medical information (eg, medications, laboratory results, and clinical notes) to fully evaluate the potential drivers of our results, such as selection bias [67], data quality factors [68], and more accurate ascertainment of the outcome (ie, unplanned readmissions).

### Conclusions

In conclusion, our analysis found that fairness metrics were useful to serve as a routine assessment to detect disparate model performance in subpopulations and to compare predictive models. However, these metrics have limited interpretability and are insufficient to inform mechanisms of bias or guide intervention planning. Further testing and demonstration will be required before using mathematical fairness measures to guide key decision-making or policy changes. Despite these limitations, demonstrating the differential model performances (eg, misclassification rates) is often the first step in recognizing potential algorithmic bias, which will be necessary as health care organizations move toward data-driven improvement in response to existing health care disparities. The potential subtle—and not so subtle—imperfections of underlying health data, analytic frameworks, and the underlying health care delivery system must be carefully considered when evaluating the potential bias that exists within predictive models. Finally, future research is required to improve the methodology of measuring algorithmic bias and to test more fairness definitions and measures (eg, calibration parity) through an operational lens. Future studies should also explore how modeling factors influence algorithmic bias (eg, how variable inclusions, weights, or scoring schemes affect the model’s differential performance). We hope that algorithmic bias assessment can be incorporated into routine model evaluation and ultimately inform meaningful actions to reduce health care disparity.

## Appendix

Multimedia Appendix 1 The LACE index.

Multimedia Appendix 2 The modified HOSPITAL score.

Multimedia Appendix 3 Income bias and hospital distribution in Maryland and Florida.

Multimedia Appendix 4 Racial and income bias measures by payer in Maryland and Florida.

## Abbreviations

AUC: area under the curve
CMS: Centers for Medicare & Medicaid Services
FNR: false negative rate
FPR: false positive rate
GEI: generalized entropy index
HCUP: Healthcare Cost and Utilization Project
SIDs: State Inpatient Databases

## Data Availability

The data sets analyzed during this study are available for a fee from the Agency for Healthcare Research and Quality Healthcare Cost and Utilization Project [37].
