# Supplementary material for: Evaluating Algorithmic Bias in 30-Day Hospital Readmission Models: Retrospective Analysis
Source: J Med Internet Res. 2024 Apr 18;26:e47125. doi: 10.2196/47125 (PMC11066744; doi:10.2196/47125)
Supplement: Multimedia Appendix 1 [file jmir_v26i1e47125_app1.docx]

**Appendix 1. LACE index**

| **Description** | **Value (LACE points)** |
| --- | --- |
| Length of stay |  |
| Less than 1 day | 0 |
| 1 day | 1 |
| 2 days | 2 |
| 3 days | 3 |
| 4–6 days | 4 |
| 7–13 days | 5 |
| ≥14 days | 7 |
| Acute or emergent admission | 3 |
| Charlson comorbidity index score |  |
| 0 | 0 |
| 1 | 1 |
| 2 | 2 |
| 3 | 3 |
| ≥4 | 5 |
| Visits to emergency department in previous 6 months |  |
| 0 | 0 |
| 1 | 1 |
| 2 | 2 |
| 3 | 3 |
| ≥4 | 4 |

*Appendix 1 Table 1. Calculating LACE index using clinical and utilization variables.*

*If LACE score >=10, it was considered “likely to be readmitted” and assigned “1” otherwise “0.” LACE score is the sum of points of the 4 LACE variables.*
