# Supplementary material for: Evaluating Algorithmic Bias in 30-Day Hospital Readmission Models: Retrospective Analysis
Source: J Med Internet Res. 2024 Apr 18;26:e47125. doi: 10.2196/47125 (PMC11066744; doi:10.2196/47125)
Supplement: Multimedia Appendix 2 [file jmir_v26i1e47125_app2.docx]

**Appendix 2: Modified HOSPITAL Score**

| **HOSPITAL Attribute** | **Points if positive** | **Adjustment** |
| --- | --- | --- |
| Low hemoglobin at discharge (<12 g/dL) | 1 | Not available in data and dropped |
| Discharge from an Oncology service | 2 |  |
| Low sodium level at discharge (<135 mEq/L) | 1 | Not available in data and dropped |
| Procedure during hospital stay (ICD10 Coded) | 1 |  |
| Index admission type urgent or emergent | 1 |  |
| Number of hospital admissions during the previous year |  |  |
| 0–1 | 0 |  |
| 2–5 | 2 |  |
| >5 | 5 |  |
| Length of stay ≥ 5 days | 2 |  |

*Appendix 2 Table 1. Calculating HOSPITAL score using clinical and utilization variables.*

|  | Original categories | Used categories after dropping two predictors | Cutoff for “likely to be readmitted” |
| --- | --- | --- | --- |
| low risk | 0-4 | 0-2 | No |
| intermediate risk | 5-6 | 3--4 | No |
| high risk | >=7 | >=5 | Yes |

*Appendix 2 Table 2. Adjusted cutoff points for HOSPITAL score.*
