# Supplementary material for: Evaluating Algorithmic Bias in 30-Day Hospital Readmission Models: Retrospective Analysis
Source: J Med Internet Res. 2024 Apr 18;26:e47125. doi: 10.2196/47125 (PMC11066744; doi:10.2196/47125)
Supplement: Multimedia Appendix 3 [file jmir_v26i1e47125_app3.docx]

**Appendix 3: Income bias and hospital distribution in MD and FL**


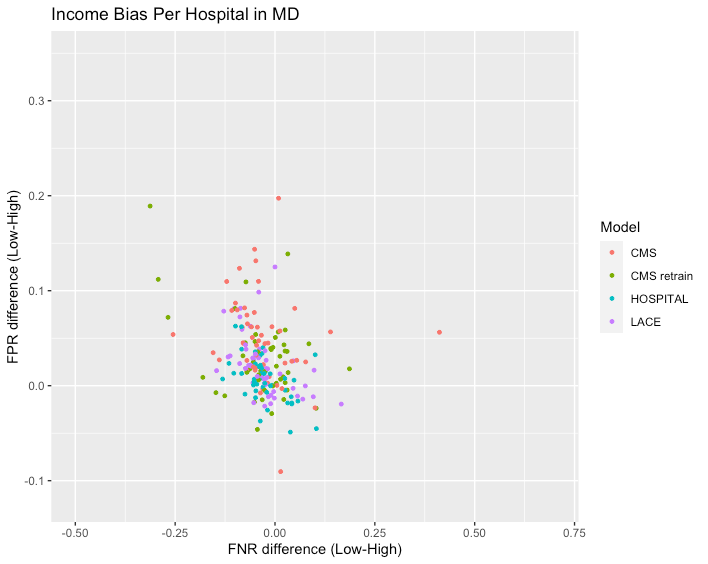


*Appendix 3 Figure 1:* *Measures of income bias and hospital distribution for Maryland*


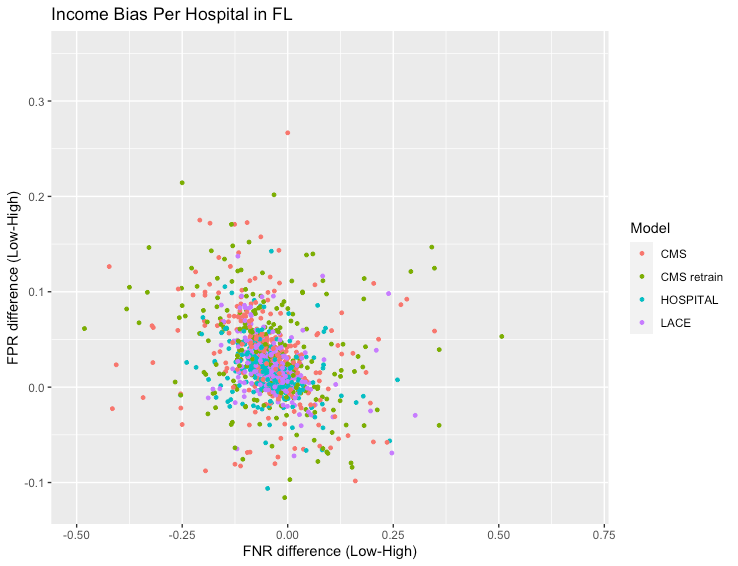


*Appendix 3 Figure 2:*  *Measures of income bias and hospital distribution for Florida*
