# Supplementary material for: Evaluating Algorithmic Bias in 30-Day Hospital Readmission Models: Retrospective Analysis
Source: J Med Internet Res. 2024 Apr 18;26:e47125. doi: 10.2196/47125 (PMC11066744; doi:10.2196/47125)
Supplement: Multimedia Appendix 4 [file jmir_v26i1e47125_app4.docx]

**Appendix 4: Racial and income bias measures by payer in MD and FL**

|  |  | Maryland | | Florida | |
| --- | --- | --- | --- | --- | --- |
| Medicare | Measure | Difference  (Black- White) | Difference (Low – Other) | Difference (Black- White) | Difference (Low – Other) |
| LACE | FNR | -0.11 | -0.06 | -0.14 | -0.05 |
| LACE | FPR | 0.07 | 0.04 | 0.09 | 0.03 |
| HOSPITAL | FNR | -0.08 | -0.06 | -0.14 | -0.06 |
| HOSPITAL | FPR | 0.04 | 0.03 | 0.06 | 0.03 |
| CMS | FNR | -0.11 | -0.06 | -0.19 | -0.05 |
| CMS | FPR | 0.13 | 0.06 | 0.16 | 0.04 |
| CMS retrain | FNR | -0.04 | -0.02 | -0.13 | -0.04 |
| CMS retrain | FPR | 0.06 | 0.04 | 0.12 | 0.04 |
| Medicaid |  | Diff (B-W) | Diff (L-O) | Diff (B-W) | Diff (L-O) |
| LACE | FNR | -0.05 | -0.04 | -0.01 | -0.01 |
| LACE | FPR | 0.01 | 0.03 | 0.00 | 0.02 |
| HOSPITAL | FNR | -0.03 | -0.05 | -0.06 | -0.04 |
| HOSPITAL | FPR | 0.00 | 0.02 | 0.00 | 0.01 |
| CMS | FNR | -0.04 | -0.03 | -0.14 | -0.03 |
| CMS | FPR | 0.03 | 0.03 | 0.11 | 0.02 |
| CMS retrain | FNR | -0.04 | -0.02 | -0.12 | -0.02 |
| CMS retrain | FPR | 0.03 | 0.02 | 0.09 | 0.03 |
| Private |  | Diff (B-W) | Diff (L-O) | Diff (B-W) | Diff (L-O) |
| LACE | FNR | -0.06 | -0.03 | -0.07 | -0.04 |
| LACE | FPR | 0.03 | 0.02 | 0.03 | 0.02 |
| HOSPITAL | FNR | -0.03 | -0.03 | -0.05 | -0.04 |
| HOSPITAL | FPR | 0.01 | 0.01 | 0.00 | 0.01 |
| CMS | FNR | -0.08 | -0.02 | -0.14 | -0.03 |
| CMS | FPR | 0.08 | 0.02 | 0.10 | 0.02 |
| CMS retrain | FNR | -0.04 | -0.01 | -0.11 | -0.03 |
| CMS retrain | FPR | 0.02 | 0.00 | 0.05 | 0.02 |
| Self-pay |  | Diff (B-W) | Diff (L-O) | Diff (B-W) | Diff (L-O) |
| LACE | FNR | -0.02 | 0.02 | 0.01 | -0.03 |
| LACE | FPR | 0.00 | 0.00 | -0.01 | 0.01 |
| HOSPITAL | FNR | 0.04 | 0.03 | -0.02 | -0.02 |
| HOSPITAL | FPR | -0.01 | 0.00 | -0.01 | 0.00 |
| CMS | FNR | -0.02 | 0.02 | -0.06 | -0.01 |
| CMS | FPR | 0.03 | 0.03 | 0.05 | 0.01 |
| CMS retrain | FNR | 0.04 | 0.09 | -0.04 | -0.01 |
| CMS retrain | FPR | 0.00 | 0.00 | 0.00 | 0.00 |
